# Discerning between Two Tuscany (Italy) Ancient Apple cultivars, ‘Rotella’ and ‘Casciana’, through Polyphenolic Fingerprint and Molecular Markers

**DOI:** 10.3390/molecules24091758

**Published:** 2019-05-07

**Authors:** Ermes Lo Piccolo, Ambra Viviani, Lucia Guidi, Damiano Remorini, Rossano Massai, Rodolfo Bernardi, Marco Landi

**Affiliations:** 1 Department of Agriculture, Food & Environment, University of Pisa, Via del Borghetto, 80–56124 Pisa, Italy; ermes.lopiccolo@gmail.com (E.L.P.); vivianiambra@outlook.it (A.V.); lucia.guidi@unipi.it (L.G.); rossano.massai@unipi.it (R.M.); rodolfo.bernardi@unipi.it (R.B.); marco.landi@agr.unipi.it (M.L.); 2 Interdepartmental Research Center Nutrafood “Nutraceuticals and Food for Health”, University of Pisa, Via del Borghetto, 80–56124 Pisa, Italy

**Keywords:** ancient cultivars, antiradical activity, apple, cluster analysis, molecular marker, organoleptic quality, pomology

## Abstract

Ancient apple cultivars usually have higher nutraceutical value than commercial ones, but in most cases their variability in pomological traits does not allow us to discriminate among them. Fruit of two Tuscany ancient apple cultivars, ‘Casciana’ and ‘Rotella’, picked from eight different orchards (four for each cultivar) were analyzed for their pomological traits, organoleptic qualities, polyphenolic profile and antiradical activity. The effectiveness of a polyphenol-based cluster analysis was compared to molecular markers (internal transcribed spacers, ITS1 and ITS2) to unequivocally discern the two apples. ‘Casciana’ and ‘Rotella’ fruit had a higher nutraceutical value than some commercial cultivars, in terms of phenolic abundance, profile and total antiradical activity. Although pedo-climatic conditions of different orchards influenced the phenolic profile of both apples, the polyphenolic discriminant analysis clearly separated the two cultivars, principally due to higher amounts of procyanidin B2, procyanidin B3 and *p*-coumaroylquinic acid in ‘Casciana’ than in ‘Rotella’ fruit. These three polyphenols can be used proficiently as biochemical markers for distinguishing the two apples when pomological traits cannot. Conversely, ITS1 and ITS2 polymorphism did not allow us to distinguish ‘Casciana’ from ‘Rotella’ fruit. Overall, the use of polyphenolic fingerprint might represent a valid tool to ensure the traceability of products with a high economic value.

## 1. Introduction

**Figure 1:**
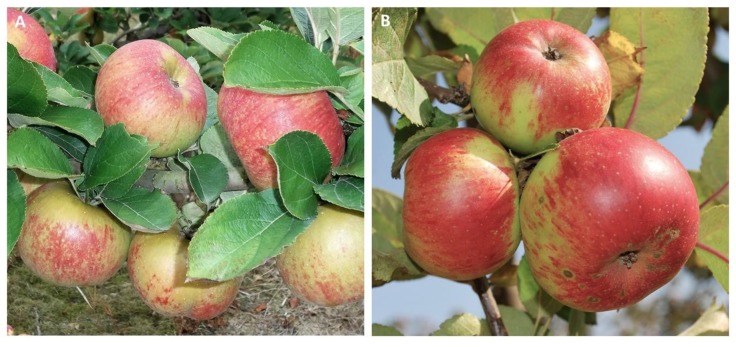
Apple fruits: from ‘Rotella’ (**A**) and ‘Casciana’ (**B**).

In Italy, the landscape complexity from ancient times has influenced the selection of numerous local apple cultivars. As long as the communities were isolated and the exchanges were limited, these cultivars had a strictly local value, but with the opening of the communities and the intensification of the exchanges, these local cultivars began to spread, mix and sometimes get confused. ‘Rotella’ and ‘Casciana’ are two ancient apple varieties typically found in Tuscany and both are listed in the germplasm bank of the Tuscany region (Regional Law N. 64, 16 November 2004; Figure 1). ‘Rotella’ is cultivated in Lunigiana and is characterized by medium-small fruits, with a slightly flattened round shape at the ends; the ‘Rotella’ apple is very tender, with a white pulp of a sweet-sour taste. The ‘Casciana’ apple is an ancient cultivar largely cultivated in Garfagnana, which is similar to the ‘Rotella’, but generally characterized by a larger and less flattened size [1]. The color of both cultivars varies with streaks from a light green to yellow, to bright red when the tree is most exposed to the sun. Even though the two varieties in most cases can be distinguished by a trained eye, in some other cases the pedo-climatic conditions influence their pomological features and/or the skin color making the visual recognition impossible, which, in turn, creates risks for local producers and paves the way for possible fraudulence.

It has been clearly demonstrated that ancient apple fruits have higher polyphenolic content if compared to some commercial cultivars, such as Golden Delicious, Fuji and Jonagold [2,3]. In addition, ancient apples are excellent sources of polyphenols, such flavonols (i.e., quercetin glycosides), flavanols (i.e., procyanidins, epicatechin and catechin), dihydrochalcones (i.e., phloridzin and phloretin 2’-O-xylosyl-glucoside) and phenolic acids (i.e., chlorogenic acid, *p*-coumaroylquinic acid, caffeic acid) [4,5,6]. It has been well established that foods rich in polyphenols have powerful cardioprotective properties and show anti-cancer activities [7,8]. In apples, these benefits are mostly related to a high flavonoid content, more specifically catechin and epicatechin in skin and pulp [7,8].

Besides the excellent nutritional value of ancient apples, the safeguard of ancient genetic material also contributes to the preservation of crop biodiversity [9,10] which is of extreme importance for fruit breeders, given that ecological systems with high biodiversity maintain a high resilience to abiotic and biotic stressors [11,12]. In a world where climate change is affecting food production, autochthonous genetic heritage can be a source of specific resistance genes in order to improve the resilience of commercial varieties to both biotic and abiotic stressors [13]. Conversely, a dramatic loss of biodiversity in apple cultivation has occurred in the last century due to the selection of a few high-yield and profitable cultivars, such Gala, Golden Delicious, Fuji, Red Delicious and Stayman [9]. In Italy, more than 70% of apples produced belong to Golden groups [14], although interest in the preservation of the autochthonous genetic heritage of fruit species is continuously growing and some neglected apple cultivars have been successfully safeguarded, rediscovered and valorized [5,6,15].

Molecular markers are an efficient way to estimate genetic diversity and determine the genetic relationships among the germplasm accessions [16,17,18,19]. Several studies using different molecular markers such as Restriction Fragment Length Polymorphisms (RFLPs) that is based on the variations in the length of DNA fragments produced by a digestion of genomic DNAs and hybridization, have been extensively used for genome comparisons [20]. Nevertheless, these types of molecular markers are highly expensive and require a high yield of DNA [21] with respect to molecular markers based on the polymerase chain reaction (PCR), including Internal Transcribed Spacers (ITSs).

Therefore, in this paper the ‘Casciana’ and ‘Rotella’ fruits were characterized in terms of pomological and organoleptic features, polyphenolic profile and total antiradical activity. In addition, we utilized polyphenol-based discriminant analyses as well as the polymorphism of ITS1 and ITS2 in the attempt to discriminate the two cultivars and to establish biochemical and/or molecular markers to unequivocally ensure the origin and traceability of both apples. ITS1 and ITS2 polymorphic sequences, which have been commonly used in phylogenetic studies [16,17,18,19,20,21,22,23,24], were also utilized to establish ITS1- and ITS2-based phylogenetic relations of ‘Casciana’ and ‘Rotella’ with other species belonging to the genus *Malus*.

## 2. Results and Discussion

### 2.1. Pomological and Organoleptic Characteristics

Apple fruit properties (weight, width max and min, solid soluble content (SSC) and titratable acidity) are listed in Table 1. Regarding pomological parameters, ‘Rotella’ fruits were generally higher in size than ‘Casciana’ ones, with larger values for width max, width min and weight. However, RBE fruits had the smallest size and the lowest values of width max and min among all accessions of both apples (46.0 g) and CPE and CBR fruits had values of width max similar to some ‘Rotella’ accession’s fruits (namely RFM and RFR). The aforementioned results indicate the impossibility of discerning the two apple cultivars by pomological features.

In terms of SSC, no significant differences between cultivars were observed. Notably, both these ancient apples are characterized by values of SSC similar to some highly appreciated commercial apples, such as ‘Fuji’, ‘Golden Delicious’ and ‘Jonagored’ [25]. Samples of CMA and CBR had higher values of TA (6.86 and 5.79 mg malic acid g^−1^, respectively), while RBE showed the lowest (2.71 mg malic acid g^−1^) among all the accessions. However, two ‘Casciana’ accession, CPR and CBE, had similar values of TA to two ‘Rotella’ types, namely RKI and RFM, highlighting that this parameter also cannot be used to distinguish ‘Casciana’ from ‘Rotella’ fruits. Titratable acidity is an important attribute to evaluate apples and it is remarkable that two ‘Casciana’ apples reach values of TA similar to those measured in ‘Granny Smith’ fruits [26,27,28].

In view of above, nor pomological or organoleptic features can be considered as affordable parameters to discriminate unequivocally between fruits belonging to the two cultivars.

### 2.2. Polyphenolic Profile, Total Antiradical Activity and Nutraceutical Attributes

In the last decades, “nutrafood” has received increasing demand from final consumers because of the strong connection between the intake of phytochemicals and the increase of human health [29]. Polyphenols are the most widely abundant secondary metabolites in the Planta kingdom and, at the same time, they represent a key source of antioxidant power for human health [7,30]. Polyphenol analysis of ancient ‘Casciana’ and ‘Rotella’ fruit showed 22 phenolic compounds belonging to four main groups (flavonols, flavanols, dihydrochalcones and phenolic acids) (Table 2). In both the cultivars, flavonols consisted of five quercetin glycosides (Q-galactoside, Q-glucoside, Q-arabinopyranoside, Q-arabinofuranoside and Q-rhamnoside). In particular, RFR and CGR had the highest content of Q-glucoside (0.44 and 0.43 µg g^−1^, respectively), whereas RBE had the highest total flavonol content (2.85 µg g^−1^ FW). Quercetin is an important dietary bioactive compound for human nutrition as it might prevent some type of cancers as well as cardiovascular diseases (CVD) [31,32]. However, its bioavailability strongly depends to the glycoside which is linked to the quercetin molecule [33]. A study conducted on rats demonstrated that Q-glucoside is rapidly absorbed by the small intestine, whereas Q-galactoside and Q-arabinopyranoside are conversely poorly absorbed [34].

Flavanols, also called flavan-3-ols, are derivates of flavans constituted by 2-phenyl-3,4-dihydro-2H-chromen-3-ol skeleton [34]. In ‘Casciana’ and ‘Rotella’ apples, they were represented by catechin, epicatechin and procyanidin B1–B4 (Table 2). Procyanidins in apple fruits belong to the B-type and are mostly constituted by epicatechin and catechin [35]. Regarding their bioavailability, only 8%–17% is absorbed by the small intestine, while the rest is metabolized by intestinal flora (especially procyanidins) of the large intestine, generating several simple phenolic compounds [34,36]. In the present work, epicatechin was the most representative compound amongst flavanols (Table 2). Epicatechin is principally absorbed by the colon (about 82%) [7], probably due to the association of epicatechin-associated fibers that can only be metabolized by the large intestinal microflora [7]. For flavanols, there is a strong inverse association between their intake, especially of catechin and epicatechin, and CVD incidence [7,8]. As these chemical compounds are relevant for human health, it is important to emphasize that ‘Casciana’ apples, independently of their accession, have higher flavanol contents than ‘Rotella’ ones, except for RBE fruit (Table 2).

The dihydrochalcones group included phlor-xyl-glucose and phloridzin that are thought to be unique in apples [37]. Evidence suggests that a large part of phloridzin and phlor-xyl-glucose are absorbed by the small intestine [37], whereas phloridzin is known to be a potent inhibitor of sodium glucose transport and, therefore, is able to modulate the postprandial blood glucose levels [38]. ‘Casciana’ and ‘Rotella accessions did not show a significant statistical differences in phlor-xyl-glucose content, whereas all accessions have similar values of phloridzin (average 17.99 µg g^−1^ FW), except for RKI which shows the lowest value (7.35 µg g^−1^ FW), therefore making both the cultivars promising sources of these compounds.

Phenolic acids represent another major group of polyphenols in apples; in the accessions of the two cultivars tested in the present experiment, 9 phenolic acids were detected: chlorogenic acid, neochlorogenic acid, cryptochlorogenic acid, *p*-coumaroyl glucose, *p*-coumaroylquinic acid, gallic acid, caffeoyl glucoside, protocatechuic acid and feruloyl glucose (Table 2). Overall, chlorogenic acid and *p*-coumaroylquinic acid are the two most representative phenolic acids, though with remarkable differences in their content in the eight apple groups. ‘Casciana’ fruit always had higher concentrations of *p*-coumaroylquinic than ‘Rotella’, independently of the orchard of origin. Chlorogenic acid is a powerful antioxidant, even though its physiological action strongly depends on its availability due to intestinal microflora that hydrolyses the molecule, giving origin to caffeic acid [39]. The hydroxycinnamic acid *p*-coumaroylquinic, in its free unconjugated form, is rapidly absorbed by the small intestine, whereas the unconjugated form is transformed by the gut microbiota in the colon [7,40]. Clinical tests conducted on *p*-coumaric acid and its conjugated forms showed antimicrobial and antiviral activities connected to its high antioxidant potential [40].

Total phenol analysis shows that two ‘Casciana’ groups, CMA and CBR, had the highest values (1120.54 and 1091.01 µg g^−1^ FW, respectively), whereas one ‘Rotella’ accession, RKI, reached the lowest value (597.71 µg g^−1^ FW). RKI was also the accession with the lowest value of phloridzin, suggesting the low nutraceutical value of fruit belonging to this accession. In any case, it should be noted that cultivars belonging to both cultivars (excluded RKI) are very rich in flavonoids and phenolic acids (flavonols and flavanols) if compared to commercial cultivars, such as Golden Delicious, Fuji and Jonagold [2,3].

**Figure 2:**
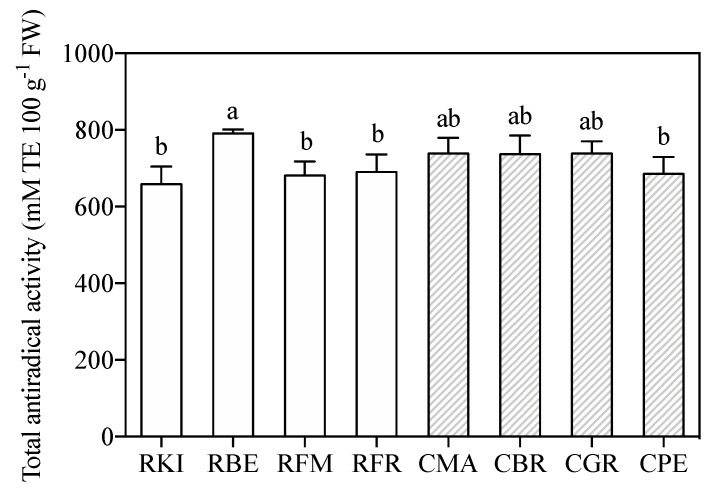
Antiradical activity determined by DPPH assay in the flesh of eight groups belonging to ‘Casciana’ and ‘Rotella’ ancient apple cultivars. Each value is the mean of three replicates ± standard deviation. The first letter of the code of each sample is indicative of the cultivar, namely ’Casciana‘ (C) or ’Rotella‘ (R). Bars with the same letter are not significantly different after a one-way ANOVA test with accession as source of variability following an LSD test (*P* = 0.05). TE: Trolox equivalent.

A high total polyphenolic content is often associated with a high antiradical activity. Values of Total Antiradical activity (TAA), measured by the DPPH radical scavenge ability (Figure 2), are found to be higher in flesh of both the ancient cultivars when compared to commercial apples [2], and RBE had the highest value (790.6 mM TE eq 100 g^−1^ FW). Furthermore, Table 3 summarizes the data obtained from the correlation analysis between the content of singular phenols versus the values of TAA. Table 3 only reports the phenols for which a significant correlation was found with TAA. According to the correlation analysis, a strong positive correlation between Q-arabinofuranoside, feruloyl glucose, caffeoyl glucoside, chlorogenic acid, Q-rhamnoside and epicatechin content and TAA was found (Table 3).

The obtained results are in agreement with other previous works which investigated the antioxidant properties of several apple cultivars [5,41,42]. To note, Tsao et al. [42] reported that flavan-3-ols, and especially procyanidins and epicatechin were the major contributors to the TAA. Although a good correlation between TAA and epicatechin was found, in our work the coefficient of correlation between procyanidins and TAA was not significant (*data not showed*). It seems therefore conceivable that values of TAA of a fruit are dependent on the whole polyphenol profile rather than on a single or a few compounds [43,44].

### 2.3. Discriminatory Analysis polyphenolic fingerprint

**Figure 3:**
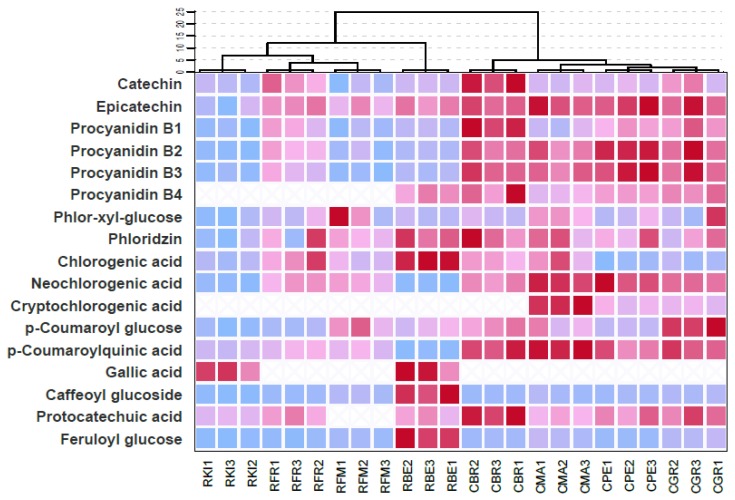
Heatmap visualization of the twenty-two phenolic compounds detected in the flesh of eight groups belonging to ‘Casciana’ and ‘Rotella’ ancient apple cultivars. The first letter of the code of each sample is indicative of the cultivar, namely ’Casciana‘ (C) or ’Rotella‘ (R). The intensity of different colors represents the phenol content (cyan = low content, red = high content). On the top side of the figure the hierarchical cluster is reported according to the phenolic profile of each group, by excluding flavonols.

Hierarchical cluster analysis (based on flavanol, dihydrochalcone and phenolic acid data matrix) and a polyphenol heatmap are shown in Figure 3. For cluster analyses, the samples (reported with all the three replicates) were separated into two homogenous groups by choosing a relatively and large safe cutting value at the linkage distance of 15. The obtained two major cluster groups fully corresponded to ‘Casciana’ and ‘Rotella’ cultivars. Through the heatmap, ‘Casciana’ and ‘Rotella’ cultivars are visually distinct. All the ‘Casciana’ accessions show higher levels of procyanidin B2, procyanidin B3 and *p*-coumaroylquinic acid than ‘Rotella’, which might be related to a constitutive preference of ‘Casciana’ apples to produce these compounds. Polyphenolic profile in apple is influenced by a plethora of environmental factors such as light, pedo-climatic conditions, agronomical practices and biotic stresses especially in the skin given that it represents the first fruit defense line [45,46]. However, the fruit responses to these external factors are strictly dependent on the interaction between the genetic background and environment and these interactions are yet to be explored in depth. Different polyphenol compositions are related to distinct apple genotypes and also the tissue-specificity of polyphenol fingerprint (i.e., in skin and flesh) is under genetic control [47]. Indeed, some authors found that apple flesh phenols and antiradical activity were linked to the different apple genotype [48,49]. Volz & McGhie [47] also showed that the variation in apple genotypes depends on polyphenol groups, in particular, chlorogenic acid, flavan-3-ols, procyanidins, dihydrochalcones and anthocyanins, whereas flavonol variation was more independent from the genotype. In our experiment, although the eight apple accessions (four for each cultivars) come from different orchards, and therefore plants were grown under different pedo-climatic and agronomical conditions, the polyphenol matrix allow us to clearly discern the two cultivars. Therefore, procyanidin B2, procyanidin B3 and *p*-coumaroylquinic seem not to be strictly influenced by environmental or agronomical conditions, and can be utilized as valid biochemical markers for a cheap and rapid methodology to discriminate and trace ‘Casciana’ and ‘Rotella’ fruits.

### 2.4. Polymorfism of ITS1 and ITS2

**Figure 4:**
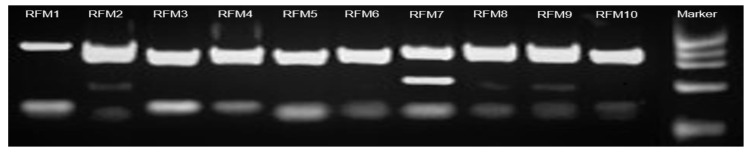
Electrophoresis of PCR colony screening of 10 colonies obtained after cloning the RFM amplified. In the right line the Marker ΦX174 DNA-HaeIII digest (Thermo Fisher Scientific, Waltham, MA, USA).

The DNA extracted from leaves of apple trees was amplified using as primers M15/M17. The amplification products after sequencing were not readable. Therefore, the PCR products were cloned into pGEM-T Easy Vector, then 12 clones were pick up from each cloning and, after PCR colony screening, the clones that showed different molecular weights, of about 900 bp, (Figure 4) were sequenced. The sequences obtained have shown the reason for the impossibility of directly sequencing the PCR product, since the primers also amplified agents used in biological control and fungi presents in the biological materials used (data not shown). The sequences were deposited in GenBank with the accession numbers MH633843–MH633854.

### 2.5. Phylogenetic Analysis Based on ITS1 and ITS2 Polymorfisms

**Figure 5:**
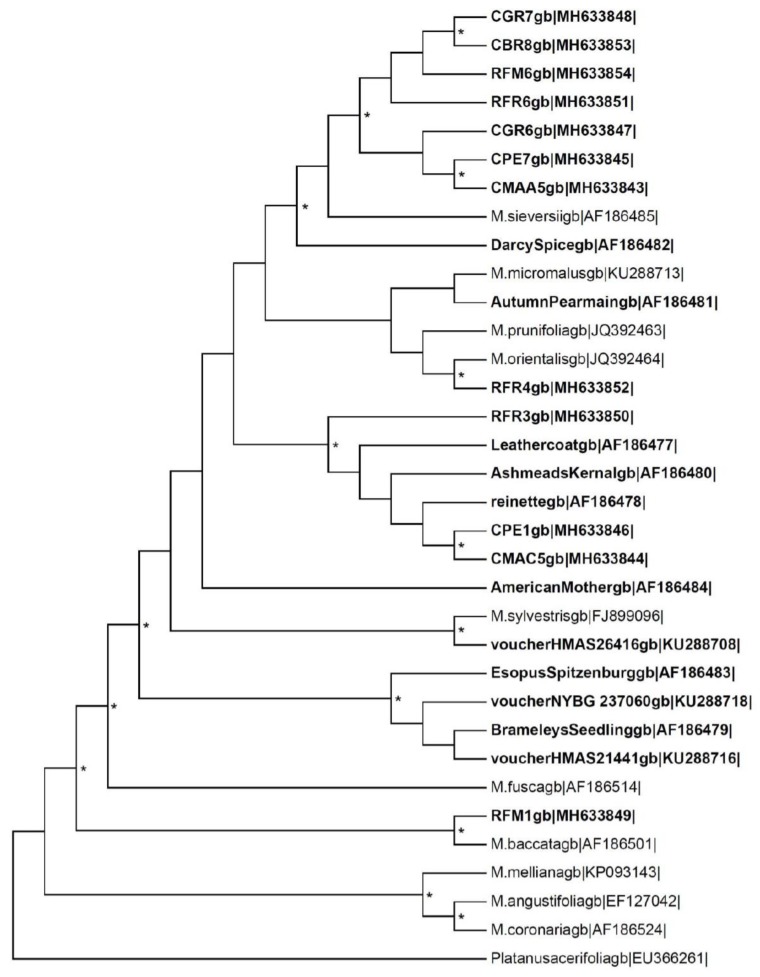
Molecular phylogenetic relationship between sequences of ‘Rotella’ and ‘Casciana’ nuclear ribosomal ITS1-5.8S-ITS2 and other sequences belonging to the genus *Malus*. The first letter of the code of each sample is indicative of the cultivar, namely ’Casciana’ (C) or ’Rotella’ (R). The taxa highlighted in bold are cultivar of *Malus domestica*. The evolutionary relationships were estimated by the statistical model Neighbor-Joining and the bootstrap was estimated with 1000 replications with the MEGA7 program. The sequence of *Platanus acerifolia* was used as outgroup. Asterisks represent a bootstrap of more than 70%.

The alignment of our sequences with sequences of *Malus* found in GeneBank allowed the construction of a phylogenetic tree using the MEGA7 program (Figure 5); as an outgroup we used the sequence of *Platanus acerifolia* found in GeneBank. The evolutionary relationships among the accessions were estimated by the statistical model Neighbor-Joining and the bootstrap was estimated with 1000 replications. The dendrogram shows that single accession of both ‘Casciana’ and ‘Rotella’ had multiple forms, such as two forms for CPE and CMA (‘Casciana’) and three forms in RFR (‘Rotella’). In addition, different forms belonging to the same accession did not form a distinct cluster but are interspersed among other accessions and also among other *Malus* species and some forms of ‘Casciana’ and ‘Rotella’ cluster together with higher level of confidence than different forms belonging to the same accession. The evolutionary relationships evaluated with these molecular markers did not enable us to distinguish ‘Casciana’ from ‘Rotella’ fruits.

## 3. Materials and Methods

### 3.1. Chemicals

Ultrapure standards used for determination of polyphenol profiles, as well as 2,2-diphenyl-1-picrydazil (DPPH), ethylenediaminetetraacetic acid (EDTA), Tris(hydroxymethyl)aminomethane hydrochloride (Tris-HCl), cetyltrimethylammonium bromide (CTAB), 2-mercaptoetanol, NaCl, were purchased from Sigma-Aldrich (Milan, Italy). Methanol, formic acid and acetronile were purchased from Carlo Erba Reagents (Cornaredo, Milan, Italy). The pGEM-T Easy Vector System were purchased by Promega (Madison, WI, USA) whereas DreamTaq and DreamTaq buffer were purchased from Thermo Fisher Scientific (Waltham, MA, USA).

### 3.2. Plant Material, Pomological and Organoleptic Properties

Fruit of ‘Casciana’ and ‘Rotella’ accessions were picked from different orchards (four for each cultivar) localized in eight different geographical places in Garfagnana and Lunigiana, (Tuscany, Italy) for ‘Casciana’ and ‘Rotella’, respectively. These were identified by a sitee code: KI (44°10′51″), BE (44°15′22″), FM (44°17′17″), FR (44°14′07″), MA (44°09′30″), BR (44°09′56″), GR (44°08′02″), PE (44°06′36″). Before the site code, every accession code was completed with the letters ‘C’ and ‘R’, which represent ‘Casciana’ and ‘Rotella’, respectively. Therefore, every code contains the apple cultivar followed by the accession name.

About 3 kg of fruits were randomly selected from different plants in each orchard at commercial maturity. Then apples were stored, one month before the analysis, in a cold chamber (4 °C and 95% of relative humidity). Three different fruits per accession were peeled and sliced with a sharping knife by removing the core portion. Little slices were cut in small portions (about 1 cm × 1 cm × 0.5 cm), mixed together and randomized in falcon tubes, frozen in liquid nitrogen and stored at −80 °C until biochemical analysis. This represented a sample replicate. Three replicates were produced and stored for biochemical analyses. Fresh weight (FW) (g) and width (mm) were calculated on eight randomly selected fruits. Solid soluble content (SSC, Brix) was analyzed on flesh juice of three randomly selected samples using a digital refractometer (refractometer Mod. 53 011, Turoni, Forli’, Italy). Titratable acidity (TA) was measured following the method reported in Landi et al. [50] on three randomly selected fruits. Fruit juice samples were diluted with deionized water (1:10) and microtritated to pH 8.0 with 0.1 NaOH and expressed as mg acid malic g^−1^.

### 3.3. Polyphenol Extraction

Flesh apple samples (about 1 g FW per sample obtained as described in Section 3.2) were homogenized with 10 mL of 70% aqueous methanol (*v/v*; 99.5% HPLC grade) by sonication for 30 min, keeping the temperature from 0 to 4 °C. After centrifugation (6000 *g* for 10 min at 4 °C), the supernatant was collected and filtered with PTFE filters (0.20 µm pore size; Sarstedt, Verona, Italy). Extracts were stored at −80 °C until analysis.

### 3.4. UPLC–MS Analysis

Phenolic profile was determined according to Assumpção et al. [51] method with few modifications. The UPLC–MS analysis was performed using an Agilent 1290 Infinity II LC system (Agilent Technologies Italia S.p.A., Cernusco sul Naviglio, Italy) consisting of a degasser, a binary pump, an autosampler, a column oven and equipped with an Agilent 6495A triple quadrupole. A C18 column, 2.1 × 50 mm, 1.8 μm (Agilent Zorbax Eclipse Plus, Santa Clara, CA, USA) was used for separation of phenolic compounds. Solvent A consisted of 0.2% formic acid in water, whereas solvent B was 0.2% formic acid in acetonitrile. The elution gradient was: 6% B (3 min), from 6 to 30% B in 11 min, from 30 to 100% B in 2 min, 100% B (2 min). The column temperature was 35 °C, the flow rate was 0.3 mL min^−1^, and the injection volume was 2 × 10^−6^ L. MS parameters employed were as follows in ESI(+): gas temp: 150 °C; gas flow: 13 L min^−1^; nebulizer: 50 psi; sheath gas heater: 350 °C; sheath gas flow: 12 L min^−1^; capillary: 3500 V, HPRF funnel: 120; LPRF funnel: 40; in ESI(−): gas temp: 150 °C; gas flow: 13 L min^−1^; nebulizer: 50 psi; sheath gas heater: 350 °C; sheath gas flow: 12 L min^−1^; capillary: 1500 V; HPRF funnel: 120; LPRF funnel: 80. For quantification, an external standard method was used. A calibration curve in at least five different concentrations from 1 to 500 μg L^−1^ was constructed for each compound analyzed and utilized to quantify each compound. Data are expressed as μg g^−1^ FW.

### 3.5. Total Antiradical Activity

Total antiradical activity (TAA), was measured using the method of Brand-Williams, Cuvelier and Berset [52]. Briefly, 10 μL of phenolic extract were added to 990 μL of a solution containing 3.12 × 10^−5^ M DPPH in methanol. The decrease in absorbance at 515 nm was measured against a blank (without extract) after reaction time of 30 min (that was preliminary optimized to observe the highest antiradical effect of the extract) using a spectrophotometer (Ultrospec 2100 pro, GE Healthcare Ltd., Chalfont St. Giles, Buckinghamshire, UK). Results (*n*=3) were expressed as percentage of reduction of the initial DPPH absorption by the extracts and expressed as mM Trolox Equivalents (TE) 100 g^−1^ FW.

### 3.6. DNA Extraction

DNA was extracted from leave of apple trees using a modified CTAB extraction method of Gawel & Jarret [53]. One hundred milligrams of leaf tissue were finely crushed using mortar and pestle and homogenized with 1 mL of CTAB extraction buffer [NaCl 1.4 M, EDTA 20 mM, Tris-HCl 100 mM, (pH 8.0), CTAB 3% (*w/v*) and 2-Mercaptoetanol 0.2% (*v/v*) in a 6:1 ratio (*v/w*)]. The mixture was recovered and transferred to 14 mL tubes and incubated for 20 min at 60 °C then extracted twice with isoamyl alcohol chloroform. After adding isopropyl alcohol to the upper phase, the DNA was precipitated at 4 °C for 1 h. The pellet obtained after centrifugation was washed with ethanol at 70% (*v/v*) and dissolved in DNase free water. The concentration of each DNA sample was measured using a WPA biowave DNA spectrophotometer (Biochrom Ltd.,Cambridge, UK), and their integrity was evaluated by agarose gel electrophoresis. The DNA was stored at −20 °C until further analysis.

### 3.7. PCR Amplification

The nuclear rDNA region, comprising the first internal transcribed spacer (ITS1), the 5.8S rRNA gene and the second internal transcribed spacer (ITS2), was amplified by the polymerase chain reaction (PCR) by using two primers, respectively complementary to the 18S and 25S rDNA near the ITS1 and ITS2 borders, M15 (5’-AAGTCGTAACAAGGTTTCCGTAGG-3’) and M17 (5’-CTTTTCCTCCGCTTATTGATATG-3’) [27].

Amplification was carried out with conventional PCR in 20 μL reactions containing 1× 10X DreamTaq Buffer and 0.5 μM of each primer, 1U of DreamTaq and 20 ng of template DNA. PCR was run in a PCR system 2700 (Applied Biosystem, Waltham, MA, USA): Thermocycling consisted of an initial denaturation step at 95 °C (5 min), which was followed of cycles for: M15/M17 (95 °C for 45 s, 60 °C for 45 s and 72 °C for 80 s), with final extension step at 72 °C (10 min).

All reactions were checked for amplification by gel electrophoresis. Amplified DNA sequences were directly cloned in pGEM-T Easy Vector System (Promega, Madison, WI, USA). Colony PCR screening was performed on individual white colonies using as primers M13 Forward and M13 Reverse. The clones that shown inserts with different weight were sequenced by automated sequencing (MWG Biotech, Ebersberg, Germany). The sequences were analyzed using BLASTN, for their identification in GeneBank.

### 3.8. Phylogenetic Analyses

The sequences are multi-aligned using CLUSTALW program [54]. The multi-alignment of the sequences of ‘Rotella’ and ‘Casciana’ accessions with the sequences of the different *Malus* species already present in the database allowed us to construct a phylogenetic tree using the MEGA7 program [55]. The evolutionary relationships were estimated by the statistical model Neighbor-Joining and the bootstrap was estimated with 1000 replications with the MEGA7 program. The sequence of *Platanus acerifolia* was used as outgroup. Asterisks represent a bootstrap higher than 70%.

### 3.9. Statistical Analysis

Data are expressed as mean ± standard deviation and are subjected to one-way ANOVA test and statistical differences among the eight groups of two apple cultivars were calculated by least significant difference (LSD) test at 95% confidence with GraphPad Software (GraphPad, La Jolla, CA, USA). Linear correlation between phenolic compounds and total antiradical activity was carried out with the same software. Hierarchical clusters were carried out using Ward’s method on normalized data matrix (using all biological replicates), in order to see similarities between cultivars by using their phenolic content. Cluster analysis (CA) was conducted using IBM SPSS Statistics 24 (IBM, New York, NY, USA). Heatmap was elaborated using GraphPad Software on the normalized polyphenol data matrix (showing all the biological replicates).

## 4. Conclusions

The current study reports new information about the nutraceutical properties of two ancient apple cultivars, ‘Rotella’ and ‘Casciana’. The polyphenolic content and the total antiradical activity found in these two ancient apple cultivars were higher than values reported for some commercial cultivars, drawing attention to the need to rediscover and re-evaluate old varieties in the attempt to find new “nutrafood” sources. When polyphenolic fingerprint was used for a cluster analyses, it allowed us to clearly separate the two cultivars and individuated three polyphenols (procyanidin B2, procyanidin B3 and *p*-coumaroylquinic acid) that were higher in ‘Casciana’ than in ‘Rotella’ accessions, independently of the orchard of origin and, therefore, of different pedo-climatic and agronomic factors. The three polyphenols mentioned above can be used proficiently as biochemical markers and their simultaneous presence can be considered as a sensitive, rapid, cheap and reliable methodology for discrimination and traceability of ‘Casciana’ and ‘Rotella’ fruit. Conversely, the molecular marker used in the present experiment, ITS1 and ITS2, did not enable us to distinguish ‘Casciana’ from ‘Rotella’ fruits.

Although the importance of these results might seem to be circumscribed at the local level, the use of chemometric parameters based on polyphenol fingerprint and the identification of valid biochemical markers is certainly of broader interest and can allow us to ensure the traceability of products with high economic value and to contrast the fraudulence phenomena.

## Figures and Tables

**Table 1:** Pomological and organoleptic characteristics of fruit of ‘Casciana’ and ‘Rotella’ apple cultivars. The first letter of the code of each sample is indicative of the cultivar, namely ‘Casciana’ (C) or ‘Rotella’ (R). Each value is the mean of eight (for weight and width) or three (for SSC and TA) replicates ± standard deviation. For each parameter, means flanked by the same letter are not significantly different after a one-way ANOVA test with accession as source of variability following an LSD test (*P* = 0.05).

Parameter	Apple Code							
	RKI	RBE	RFM	RFR	CMA	CBR	CGR	CPE
Weight (g)	132.81 ± 15.79 ^a^	46.00 ± 3.42 ^f^	110.64 ± 6.54 ^c^	124.43 ± 6.04 ^b^	69.05 ± 10.58 ^e^	94.73 ± 5.16 ^d^	69.33 ± 5.24 ^e^	89.36 ± 5.26 ^d^
Width min (mm)	70.02 ± 3.48 ^a^	47.48 ± 3.14 ^d^	66.07 ± 2.14 ^ab^	66.67 ± 2.10 ^ab^	53.80 ± 2.47 ^c^	60.86 ± 2.95 ^b^	57.66 ± 3.16 ^bc^	60.01 ± 4.12 ^b^
Width max (mm)	74.93 ± 5.11 ^a^	55.08 ± 3.33 ^c^	71.26 ± 8.50 ^a^	71.11 ± 3.31 ^a^	57.50 ± 1.34 ^c^	64.46 ± 2.85 ^b^	55.31 ± 2.29 ^c^	68.22 ± 2.82 ^ab^
SSC (°Brix)	15.60 ± 0.68	17.20 ± 1.40	15.40 ± 0.58	17.70 ± 0.98	16.80 ± 1.56	16.60 ± 1.10	14.80 ± 0.67	17.70 ± 1.90
Titratable acidity (mg malic acid g^−1^ FW)	4.28 ± 0.27 ^c^	2.71 ± 0.32 ^d^	3.83 ±0.82 ^c^	4.50 ± 0.23 ^c^	6.86 ± 0.29 ^a^	5.79 ± 0.59 ^b^	3.85 ± 0.39 ^c^	3.92 ± 0.39 ^c^

**Table 2:** Polyphenols profile (μg g^−1^ FW) of fruit of ‘Casciana’ and ‘Rotella’ apple cultivars. The first letter of the code of each sample is indicative of the cultivar, namely ’Casciana‘ (C) or ’Rotella‘ (R). Each value is the mean of three replicates ± standard deviation. For each phenol, means flanked by the same letter are not significantly different after a one-way ANOVA test with cultivars as source of variability following an LSD test (*P* = 0.05).

Polyphenols	Apple Code							
	RKI	RBE	RFM	RFR	CMA	CBR	CGR	CPE
**Flavonols**								
Q-galactoside	0.03 ± 0.005 ^c^	0.07 ± 0.01 ^b^	0.04 ± 0.005 ^c^	0.05 ± 0.01 ^c^	0.05 ± 0.005 ^c^	0.08 ± 0.004 ^b^	0.14 ± 0.02 ^a^	0.04 ± 0.01 ^c^
Q-glucoside	0.17 ± 0.01 ^b^	0.35 ± 0.08 ^ab^	0.27 ± 0.04 ^b^	0.44 ± 0.04 ^a^	0.29 ± 0.01 ^b^	0.36 ± 0.15 ^ab^	0.43 ± 0.07 ^a^	0.26 ± 0.05 ^b^
Q-arabinopyranoside	0.07 ±0.006 ^c^	0.14 ± 0.03 ^b^	0.14 ± 0.02 ^b^	0.20 ± 0.04 ^a^	0.11 ± 0.002 ^bc^	0.12 ± 0.03 ^bc^	0.19 ± 0.01 ^ab^	0.14 ± 0.06 ^b^
Q-arabinofuranoside	0.08 ± 0.008 ^c^	0.35 ± 0.01 ^a^	0.20 ± 0.02 ^b^	0.29 ± 0.03 ^ab^	0.16 ± 0.01 ^bc^	0.24 ± 0.12 ^b^	0.31 ± 0.02 ^ab^	0.17 ± 0.07 ^b^
Q-rhamnoside	0.49 ± 0.054 ^d^	1.94 ± 0.12 ^a^	0.87 ± 0.07 ^c^	1.40 ± 0.35 ^b^	0.85 ± 0.04 ^c^	0.88 ± 0.20 ^c^	1.10 ± 0.18 ^c^	1.26 ± 0.11 ^bc^
**Total**	0.84 ± 0.06 ^e^	2.85 ± 0.22 ^a^	1.52 ± 0.15 ^cd^	2.38 ± 0.41 ^b^	1.46 ± 0.05 ^d^	1.68 ± 0.49 ^cd^	2.17 ± 0.27 ^bc^	1.87 ± 0.30 ^c^
**Flavanols**								
Catechin	37.07 ± 2.62 ^cd^	42.42 ± 0.89 ^c^	31.92 ± 7.57 ^d^	61.53 ± 6.93 ^b^	44.63 ± 2.27 ^c^	79.36 ± 6.42 ^a^	55.82 ± 11.06 ^bc^	46.09 ± 2.43 ^c^
Epicatechin	191.08 ± 39.88 ^c^	307.65 ± 13.09 ^b^	272.16 ± 29.17 ^b^	305.63 ± 10.26 ^b^	354.08 ± 28.82 ^ab^	336.45 ± 14.31 ^ab^	345.65 ± 35.30 ^ab^	361.40 ± 29.53 ^a^
Procyanidin B1	5.47 ± 0.40 ^e^	6.99 ± 0.34 ^de^	6.01 ± 0.92 ^e^	9.39 ± 0.95 ^c^	7.55 ± 0.87 ^d^	13.11 ± 0.79 ^a^	10.72 ± 0.96 ^b^	9.91 ± 0.46 ^bc^
Procyanidin B2	4.62 ± 0.17 ^e^	5.97 ± 0.21 ^d^	5.68 ± 1.12 ^de^	8.94 ± 0.40 ^c^	10.51 ± 0.92 ^b^	10.75 ± 0.64 ^b^	11.43 ± 1.50 ^b^	12.52 ± 0.18 ^a^
Procyanidin B3	43.53 ± 3.53 ^e^	55.06 ± 2.41 ^d^	42.35 ± 3.70 ^e^	78.08 ± 9.45 ^c^	105.19 ± 6.36 ^b^	111.87 ± 7.04 ^b^	112.99 ± 14.93 ^ab^	123.93 ± 10.51 ^a^
Procyanidin B4	-	1.03 ± 0.11 ^b^	-	-	0.72 ± 0.08 ^c^	1.30 ± 0.37 ^a^	1.11 ± 0.10 ^ab^	0.97 ± 0.01 ^b^
**Total**	281.77 ± 35.03 ^d^	419.12 ± 13.96 ^b^	358.12 ± 41.02 ^c^	463.57 ± 9.26 ^b^	522.68 ± 31.97 ^a^	552,84 ± 26.42 ^a^	537.72 ± 60.36 ^a^	554.82 ± 39.53 ^a^
**Dihydrochalcones**								
Phlor-xyl-glucose	29.78 ± 7.35	40.34 ± 3.05	63.05 ± 26.89	46.69 ± 6.86	61.50 ± 3.56	44.64 ± 4.80	52.30 ± 25.58	45.59 ± 9.06
Phloridzin	7.35 ± 2.67 ^c^	21.35 ± 1.96 ^a^	15.58 ± 1.42 ^ab^	15.39 ± 8.11 ^b^	18.64 ± 4.25 ^ab^	21.31 ± 4.37 ^a^	16.17 ± 4.47 ^ab^	17.49 ± 3.94 ^ab^
**Total**	37.13 ± 8.44	61.69 ± 4.98	78.63 ± 28.31	62.08 ± 14.91	80.14 ± 7.81	65.95 ± 9.17	68.47 ± 28.72	63.08 ± 12.67
**Phenolic acids**								
Chlorogenic acid	195.97 ± 13.84 ^d^	403.31 ± 10.80 ^a^	246.08 ± 20.94 ^c^	324.66 ± 42.79 ^b^	309.55 ± 51.13 ^b^	293.39 ± 12.96 ^b^	191.61 ± 20.97 ^d^	166.47 ± 13.33 ^d^
Neochlorogenic acid	7.20 ± 0.56 ^e^	6.78 ± 0.17 ^e^	17.96 ± 1.62 ^d^	19.05 ± 1.49 ^cd^	26.09 ± 1.21 ^a^	20.42 ± 0.93 ^c^	22.27 ± 0.38 ^b^	25.63 ± 2.77 ^a^
Cryptochlorogenic acid	-	-	-	-	0.71 ± 0.05 ^a^	-	0.32 ± 0.03 ^b^	0.34 ± 0.05 ^b^
*p* -Coumaroyl glucose	1.05 ± 0.14 ^e^	2.00 ± 0.24 ^c^	2.51 ± 0.44 ^b^	1.31 ± 0.10 ^de^	2.23 ± 0.44 ^bc^	2.58 ± 0.19 ^b^	3.35 ± 0.23 ^a^	1.60 ± 0.05 ^d^
*p* -Coumaroylquinic acid	64.96 ± 5.10 ^e^	27.52 ± 2.12 ^f^	80.82 ± 8.98 ^d^	86.21 ± 9.01 ^d^	154.50 ± 5.51 ^a^	138.28 ± 11.86 ^b^	131.00 ± 9.13 ^b^	118.79 ± 13.18 ^c^
Gallic acid	0.03 ± 0.005	0.03 ± 0.01	-	-	-	-	-	-
Caffeoyl glucoside	0.10 ± 0.01 ^c^	0.93 ± 0.10 ^a^	0.25 ± 0.04 ^b^	0.16 ± 0.01 ^c^	0.22 ± 0.05 ^bc^	0.17 ± 0.01 ^c^	0.25 ± 0.02 ^b^	0.20 ± 0.02 ^bc^
Protocatechuic acid	0.04 ± 0.002 ^d^	0.06 ± 0.01 ^c^	-	0.06 ± 0.01 ^c^	0.05 ± 0.005 ^c^	0.10 ± 0.01 ^a^	0.08 ± 0.01 ^b^	0.07 ± 0.01 ^bc^
Feruloyl glucose	8.64 ± 0.90 ^d^	75.47 ± 6.61 ^a^	15.56 ± 1.91 ^c^	10.47 ± 2.18 ^d^	22.91 ± 3.88 ^b^	15.60 ± 1.11 ^c^	23.68 ± 2.46 ^b^	12.63 ± 0.21 ^cd^
**Total**	277.97 ± 19.55 ^e^	516.10 ± 6.55 ^a^	363,18 ± 22.63 ^cd^	441.92 ± 53.28 ^b^	516.26 ± 48.26 ^a^	470.54 ± 3.72 ^b^	372.56 ± 28.43 ^c^	325,73 ± 6,64 ^d^
**Total polyphenols**	**597.71 ± 61.94 ^d^**	**999.76 ± 11.81 ^b^**	**801.45 ± 59.47 ^c^**	**969.95 ± 60.13 ^b^**	**1120.54 ± 63.53 ^a^**	**1091.01 ± 37.78 ^a^**	**980.92 ± 23.74 ^b^**	**945.50 ± 53.04 ^b^**

**Table 3:** Correlation coefficients (r) between selected phenols and total antiradical activity of fruit of ‘Casciana’ and ‘Rotella’ apple accessions. Table only reports the phenols for which a significant correlation was found with the total antiradical activity (*: *P* < 0.05, **: *P* < 0.01; ***: *P* < 0.001).

Phenol	Correlation
Caffeoyl glucoside	0.61 **
Chlorogenic acid	0.58 **
Epicatechin	0.51 *
Feruloyl glucose	0.66 ***
Q-arabinofuranoside	0.68 ***
Q-rhamnoside	0.58 **
